# Lateral orbital wall reconstruction after basal cell carcinoma penetration—Case report

**DOI:** 10.3389/fsurg.2022.925824

**Published:** 2022-09-05

**Authors:** Bruno Popić, Andrijana Kopić, Dubravka Holik, Kristijan Dinjar, Vlatko Kopić, Marko Matijević, Fran Popić

**Affiliations:** ^1^ Department of Maxillofacial and Oral Surgery, Osijek University Hospital Centre, Osijek, Croatia; ^2^ Department of Ophthalmology, Osijek University Hospital Centre, Osijek, Croatia; ^3^ School of Medicine, Josip Juraj Strossmayer University of Osijek, Osijek, Croatia; ^4^ Faculty of Dental Medicine and Health, Josip Juraj Strossmayer University of Osijek, Osijek, Croatia; ^5^ Dental Practice, Community Healthcare Center of Osijek-Baranja County, Osijek, Croatia

**Keywords:** lateral orbital wall, basal cell carcinoma, penetration, reconstruction, titanium mesh

## Abstract

Advanced periorbital basal cell carcinomas may necessitate orbital exenteration and consequent vision loss, which significantly reduces patients’ life quality. Orbital reconstruction is a demanding surgical procedure due to the complex orbital anatomy and vital structures located in the orbit. In this report, we presented an 83-year-old patient with advanced basal cell carcinoma that had expanded into the orbit. An orbitotomy was performed to remove the tumor completely while preserving the eye function. Orbital reconstruction was performed by a standard surgical method using a titanium mesh modeled according to a natural phantom skull. This maintained the eye function and achieved satisfactory facial esthetics.

## Introduction

Basal cell carcinoma (BCC) is the most common malignant skin tumor and accounts for approximately 90% skin cancers (1, 2). The incidence of skin malignancy with orbital invasion is 2%–4% (3, 4). BCC most commonly occurs in the lower eyelid and medial canthus (5). Initially, BCC shows no signs of orbital penetration, although tumor mass is always palpable (6). With disease progression, BCC erodes the bone. Penetration of the periorbita and the involvement of extraocular muscles can restrict eye movement and lead to eyeball dislocation (7). Computed tomography (CT) is a suitable imaging technique for bone destruction diagnosis, and magnetic resonance imaging is reliable for detecting soft tissue orbital invasion (8). In the case of diagnostically confirmed invasion of the malignancy into the orbit, therapy planning requires the collaboration of a multidisciplinary team (2). When planning the surgical procedure, it is necessary to take into the account biological behavior of the tumor, histological findings, and the possibility of perineural invasion, which requires extended edges for surgical excision (1, 2). After orbital exenteration, recurrences occur in about 5% of cases. Inadequate BCC treatment leads to orbital invasion and, consequently, a greater number of orbital exenterations (9), which was confirmed by Bartley et al. in 80% of cases with failed first treatment (surgery or/and radiotherapy) (10).

In this case report, we present a case where we performed lateral orbital wall reconstruction with a titanium mesh previously adapted and modeled using a natural phantom skull and avoided orbital exenteration. Reconstruction of orbital wall defects after tumor resection due to complex anatomy is also a challenge even for experienced maxillofacial surgeons.

## Case report

An 83-year-old patient was admitted in 2018 to our Department of Maxillofacial and Oral Surgery, University Hospital Center Osijek, because of a tumor in the frontozygomatic region on the right side. In 2010, the patient was treated in the surgical department of another institution. According to the pathohistological findings, it was a skin basal cell carcinoma. Due to the irregularity of the preparation, it was not possible to assess the status of the edges of the preparation, so the patient was referred to our department. He neglected that recommendation for next eight years and did not perform the examination in our department. In October 2018, the patient was examined by an ophthalmologist, who performed lower eyelid ectropion correction twice and referred the patient to our department now due to suspected recurrence of the tumor frontotemporally in the right side.

Examination revealed a serious impaired general condition of the patient (Parkinson's disease, moving difficulties, prostate hyperplasia, and poorly regulated hypertension). In the local status, we found a tumorous change of 3 cm × 3 cm in the frontozygomatic region fixed to the right on the base, right eye lower eyelid ectropion, while the regional lymph nodes were not palpable.

**Figure 1:**
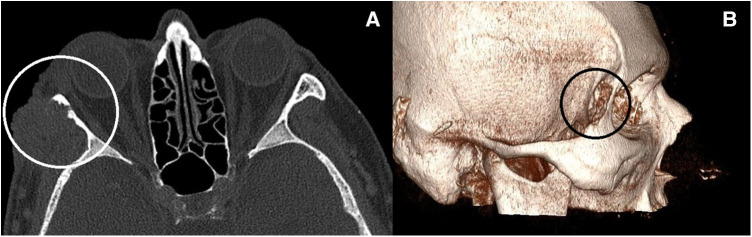
(**A,B**) Orbital CT scan—tumor eroding the bone.

During the preoperative preparation, a native and postcontrast orbital CT was performed and a lobulated solid expansive formation of the zygomatic region on the right (2.5 cm × 2.3 cm ×  2.1 cm) was determined, which infiltrated and eroded the frontozygomatic extension on the right side 1.8 cm × 1.7 cm long (Figures 1A,B).

In the infratemporal space on the right, a solid formation size 1.5 cm × 1.0 cm was differentiated without right eye bulbus infiltration. Tumor had two lobes, one lobe that affected the skin and was fixed to the eroded bone and the other part that was in the infratemporal cavity and penetrated into the orbit through the lateral wall of the orbit, so the tumor was folded over the lateral edge of the orbit.

**Figure 2:**
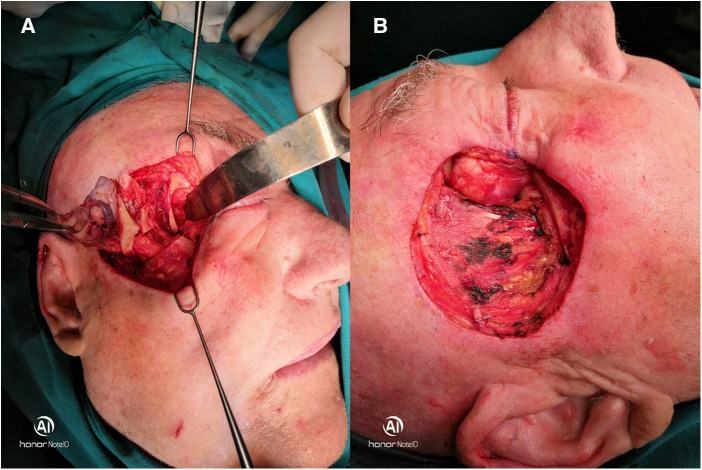
(**A,B**) Surgical approach with the resection of the lateral orbital wall and completely removed tumor with a preserved eyeball.

Based on the CT findings, we decided to operate the tumor with resection of the lateral orbital wall while preserving the eyeball. With written informed consent of the patient, under general anesthesia, an incision was made in the skin and subcutaneous tissue around the tumor, with adequate surgical margins (Figure 2A). During the operation, the tumor was removed completely with the resection of the lateral orbital wall, part of the body and the beginning of the arch of the zygomatic bone, the associated periosteum, orbital fat, and the lateral canthal ligament. Right eyeball and ocular muscles remained preserved (Figure 2B).

**Figure 3:**
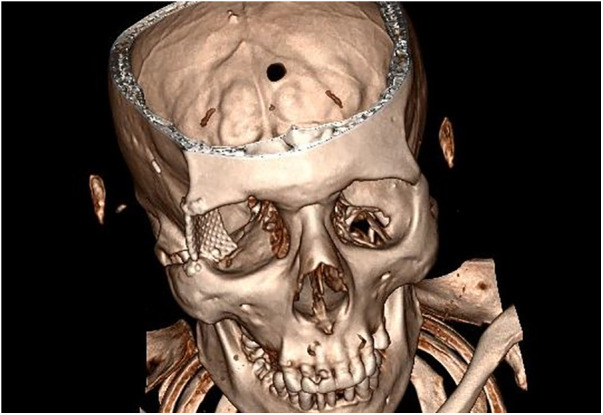
Natural phantom skull model for a titanium mesh.

Bone defect of the lateral orbital wall was reconstructed using a titanium mesh previously adapted and modeled using a natural phantom skull (Figure 3).

**Figure 4:**
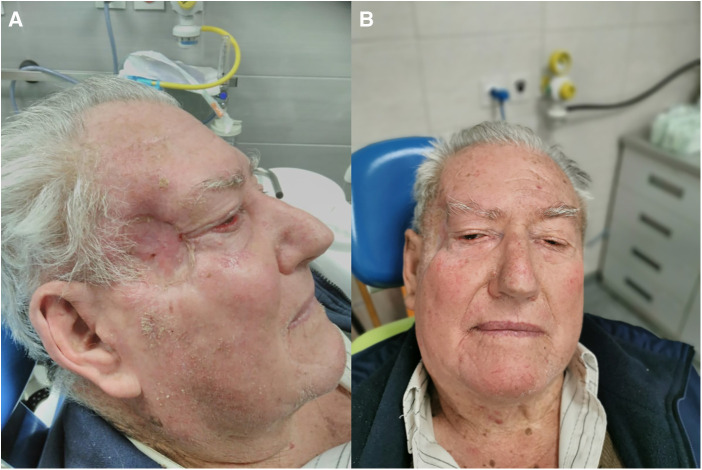
(**A,B**) Eyelids and mild face asymmetry after the surgery.

Once the mesh was fixed in a satisfactory position, a portion of the temporalis muscle and temporal fascia was pulled over it. The entire defect was then covered with a Thiersch skin graft taken from the right thigh. The skin graft was fixed with sutures to the surrounding skin with compression with a cotton ball with Vaseline gauze. The site of Thiersch transplant on the right thigh was covered with a Kaltostat, which was removed after three weeks. Compression of the skin graft and sutures were removed after one week. It should be emphasized that the patient did not agree to local skin flaps, which was good because a thin skin graft allowed more adequate control of the occurrence of possible tumor recurrence. The patient was controlled regularly for two years by a maxillofacial surgeon and an ophthalmologist. A control CT scan was performed without recurrence of the tumor, eye motility was in order, as was the function of eyelids closing (Figure 4). Mild asymmetry of the right side of the face was left behind.

## Discussion

Malignant primary or secondary tumors that penetrate the orbit from the surrounding skin, maxillary sinus, or neurocranium require surgical treatment. The incidence of malignant tumors of the skin of the periorbital region is constantly increasing, and accordingly, an increased number of indications for orbital exenteration are expected. Kesting et al. in their study reported that 99.3% of patients underwent orbital exenteration due to a malignant tumor, and in 23.8% of cases, the cause was the progression of skin cancer into the orbit (11). Usually, orbital exenteration is performed when the malignant process affects the periorbital tissue, external eye muscles, orbital apex, and in case of vision loss. Orbital reconstruction is the most challenging part of midface surgery, especially in preserved eyeballs, and aims to restore the contours, volume of the orbit, and position of the eyeball. The decision on reconstruction is made taking into account the available materials and techniques of reconstruction and always in agreement with the patient (12). In the case of a preserved eyeball, functional and esthetic defects are common, and they include enophthalmos, hypophthalmus, eyelid malposition, epiphora, and diplopia. Subsequent surgical corrections of structural disorders are often unsuccessful, so it is necessary to choose the optimal primary method of orbital reconstruction whenever it is possible. During orbital reconstruction, it is important to keep the eyelids in a normal position to protect the eye from external influences (13). A review of the literature suggests various approaches to midface reconstruction and the focus is on midface reconstruction as a whole, but a structural approach to the orbit is a bit lacking. Thus, there is no consensus on the classification of orbital defects and consequently no standardized reconstructions (14). Titanium mesh implants, bone grafts, or polyether-ether-ketone implants can be used to reconstruct the orbit. The success of the reconstruction depends not only on the type of material used for the orbital reconstruction but also on the possibility of visualizing the bone defect (15). Kim et al., like Ellis and Messo, reported that titanium mesh is an excellent alloplastic material for orbital reconstruction (16, 17). The low rejection rate, extrusion and infection, is attributed to the characteristic of titanium to cause an inflammatory and fibrous response of the surrounding tissue (18). The use of individual titanium meshes shortens the duration of the operation because it is possible to position the mesh better without unnecessary multiple removal and placement of implants, which further traumatizes the soft tissue and sensitive structures of the orbit (18). Computer-assisted design/computer-aided fabrication (CAD/CAM) reconstruction of individual implants is an effective procedure that allows for very precise reconstruction of the complex orbital anatomy. CAD/CAM 3D planning and customized implants are now considered the gold standard in most centers (19–21).

## Data Availability

The original contributions presented in the study are included in the article/Supplementary Material, further inquiries can be directed to the corresponding author.
